# *Iacta Alea Est*: The Inexorable Advance of Tofacitinib in the Treatment of Dermatomyositis-Associated Rapidly Progressive Interstitial Lung Disease. A Case Report

**DOI:** 10.3389/fphar.2020.585761

**Published:** 2020-12-15

**Authors:** Walter Conca, Ihab Weheba, Mohei-Eldin Abouzied, Abeer Abdelsayed, Yousif Aleyouni, Eid Al‐Mutairy, Nasir Bakshi, Mohammad Khalid

**Affiliations:** ^1^ Department of Medicine, King Faisal Specialist Hospital and Research Centre, Riyadh, Saudi Arabia; ^2^ College of Medicine, Alfaisal University, Riyadh, Saudi Arabia; ^3^ Department of Pulmonary Medicine, National Research Centre, Cairo, Egypt; ^4^ Department of Radiology, King Faisal Specialist Hospital and Research Centre, Riyadh, Saudi Arabia; ^5^ Department of Pulmonary Medicine, Ain Shams University, Cairo, Egypt; ^6^ Department of Dermatology, King Faisal Specialist Hospital and Research Centre, Riyadh, Saudi Arabia; ^7^ Department of Pathology and Laboratory Medicine, King Faisal Specialist Hospital and Research Centre, Riyadh, Saudi Arabia

**Keywords:** tofacitinib, dermatomyositis, anti Jo-1 antibody, rapidly progressive interstitial lung disease, ferritin, natural killer cell, nintedanib, case report

## Abstract

Rapidly progressive interstitial lung disease is typically associated with clinically amyopathic dermatomyositis and the anti-melanoma differentiation associated gene 5 antibody, a condition with high mortality and resistance to classic immunosuppression. Recent reports have described the efficacy of the Janus kinase inhibitor tofacitinib in the treatment of rapidly progressive interstitial lung disease in anti-melanoma differentiation associated gene 5 antibody-positive clinically amyopathic dermatomyositis. It is uncertain, however, whether tofacitinib alters the course of rapidly progressive interstitial lung disease in other variants of dermatomyositis that are unrelated to the anti-melanoma differentiation associated gene 5 antibody and whether the early addition of the anti-fibrotic tyrosine kinase inhibitor nintedanib interferes with the development of fibrosis. To answer these questions, we present and discuss the case of an elderly woman who presented with a flare of dermatomyositis *sine* myositis. Based upon the detection of anti-Jo-1 antibodies and the absence of anti-melanoma differentiation associated gene 5 antibodies, anti-synthetase syndrome was diagnosed. While the cutaneous manifestations quickly resolved with prednisone, azathioprine and tacrolimus, the respiratory function paradoxically and rapidly deteriorated, and invoked the use of tofacitinib. Markedly raised ferritin levels and a severe numerical deficiency of circulating natural killer cells paralleled the acute lung inflammation, which was reflected by ^18^F-fluorodeoxyglucose hypermetabolism on positron emission tomography/CT. Tofacitinib lead to a prompt clinical recovery, with a reduction in oxygen requirement, correction of hyperferritinemia, reversal of the natural killer cell deficiency, and a decrease in ^18^F-fluorodeoxyglucose uptake in the affected lung segments. Subsequently, nintedanib was added at a point in time when inflammation subsided. Apart from cytomegalovirus reactivation no adverse events occurred. In conclusion, tofacitinib reversed the pronounced inflammatory component of anti-Jo-1 antibody-positive, anti-melanoma differentiation associated gene 5 antibody-negative rapidly progressive interstitial lung disease, confirming that Janus kinase signaling pathways are critically involved in the pathogenesis of rapidly progressive interstitial lung disease, apparently independently of the targeted autoantigen. Although some improvement in pulmonary function was observed, it seems premature to conclusively judge on reversibility or prevention of pulmonary fibrosis by pairing both kinase inhibitors for which an extended follow-up and ideally, prospective and controlled studies are needed.

## Introduction

Tofacitinib, a non-selective Janus kinase (JAK) inhibitor introduced first in the treatment of rheumatoid arthritis (Lundquist et al., 2014), has recently been shown to alter the grave prognosis of rapidly progressive (RP) interstitial lung disease (ILD) in anti-melanoma differentiation associated gene 5 (MDA5) antibody-positive clinically amyopathic (CA) dermatomyositis (DM) in patients from Japan and China (Kurasawa et al., 2018; Chen et al., 2019; Kato et al., 2019). Conventional immunosuppressive therapy is inadequate to halt the progression of an often fulminant ILD, and therefore, the positive outcome resulting from interference with JAK-dependent signaling pathways has the power to change the management of this dreadful complication of DM. Beside the clinical improvement correction of marked hyperferritinemia, an ominous biomarker of disease severity, was reported (Gono et al., 2012; Osawa et al., 2018). With regard to the fibrotic component of RPILD, pirfenidone delays the progression of lung fibrosis (Li et al., 2016), and nintedanib, an anti-fibrotic tyrosine kinase inhibitor, decreases the rate of decline in forced vital capacity (FVC) in various ILDs (Richeldi et al., 2014; Wollin et al., 2015; Distler et al., 2019; Flaherty et al., 2019). Therefore, adding nintedanib early in RPILD has the potential to interfere with the development of fibrosis.

RPILD typically occurs in association with anti-MDA5 antibodies, whereas ILD with insidious onset, a pattern of non-specific interstitial pneumonia (NSIP) and adequate response to immunosuppression is found in the context of anti-aminoacyl-tRNA-synthetase antibodies, most frequently anti-Jo-1 (histidyl-tRNA synthetase) (Sato et al., 2009; Koreeda et al., 2010; Chen et al., 2013; Gasparotto et al., 2019). Intriguingly, the epitopes targeted by these autoantibodies reside in two unrelated cytoplasmic proteins: MDA5 represents a helicase that binds double-stranded RNA and functions as an antiviral pattern recognition receptor (Reikine et al., 2014), whereas histidyl-tRNA-synthetase generates histidyl-tRNA, which incorporates histidine into polypeptide chains (Freist et al., 1999). Here we describe an exceptional case of a fulminant organ-specific autoimmune attack in the presence of anti-Jo-1 antibodies, that was clinically indistinguishable from anti-MDA5 antibody-associated lung injury. Tofacitinib swiftly reduced the pulmonary inflammation, indicating that JAK signaling is key to the pathogenesis of DM-associated RPILD. With the mounting evidence of the efficacy of tofacitinb in the treatment of severe cutaneous manifestations, including *calcinosis*, in DM this drug could soon advance to an indispensable tool in the management of serious skin and critical lung disease (Paik and Christopher-Stine, 2017; Moghadam-Kia et al., 2019; Sabbagh et al., 2019; Wendel et al., 2019).

## Patient Information

A 74-year old woman of Arab ethnicity living in the Central Region of Saudi Arabia presented to the outpatient clinic on October 1, 2019 with a two-week history of severe fatigue, a rash involving the dorsal aspects of the fingers, palms, cheeks and forehead, and a dry, irritating cough. A diagnosis of DM had been established earlier in 2011 when the disease manifested with Gottron’s sign accompanied by a single punched out skin ulcer over the dorsal aspect of the second metacarpophalangeal joint (MCP) of the left hand, periungual hyperkeratosis and proximal nailfold telangiectasia. At that time, there was no muscle weakness and creatine kinase (CK) levels were normal. However, MRI demonstrated symmetric muscle edema in serratus anterior, subscapularis, gluteus maximus and biceps femoris. In an outside laboratory, anti-Mi-2 antibodies were detected, and therefore the DM was classified as hypomyopathic. There was no lung involvement and four months later the patient developed *Pneumocystis jiroveci* pneumonia (PCP) in the presence of marked lymphopenia, probably drug-induced as the patient was on high-dose corticosteroid and methotrexate. She recovered from the infection and maintained remission with prednisone 5 mg oral (p.o.) on alternate days and azathioprine 50 mg/day p.o. until September 2019 when the disease recurred.

## Clinical Findings

On presentation, vital signs were as follows: temperature 36.8°C, heart rate 98 beats/min, respiratory rate 20/min, blood pressure 133/80 mmHg and 96% oxygen (O_2_) saturation in room air. A pathognomonic patchy, dusky rash was present not only on the dorsal aspect of the MCP and proximal interphalangeal joints, but also on the palms, cheeks and forehead, without ulcerations. There was proximal nailfold telangiectasia and periungual erythema, but neither digital fissuring, heliotrope erythema, shawl sign, V-sign, holster sign, Raynaud’s phenomenon nor joint pain was present. The muscle power was normal. She had short bouts of a non-productive cough without hemoptysis. Chest examination revealed a normal expansion and on auscultation fine crepitations over both lung bases were noted.

## Diagnostic Assessment

### Laboratory Results

Table 1 shows hematologic, biochemical and immunologic variables at different points in time: on presentation (October 1, 2019), hospital admission (October 21), initiation of tofacitinib (November 1) and nintedanib (November 18), eight weeks (December 25) and 18 weeks (March 8, 2020) after starting tofacitinib.

**TABLE 1:** Laboratory data on presentation (October 1, 2019), admission (October 21), initiation of tofacitinib (November 1), nintedanib (November 18), eight weeks (December 25) and 18 weeks (March 8, 2020) after starting tofacitinib. Measurements were performed on Sysmex XN-9000 for hematologic and Roche/Hitachi cobas^®^ c for other variables. Flow cytometry of lymphocytes was done on a BD FACSCanto™ II flow cytometer on the same dates, except for October 23 and 30, 2019.

Variable	October 1, 2019	October 21	November 1	November 18	December 25	March 8, 2020	References range
Hemoglobin (g/L)	122	123	124	98	108	115	110–160
WBC (×10^9^/L)	6.5	12.05	6.8	7.6	10.2	7.3	3.9–11
Neutrophils (×10^9^/L)	5.1	11.4	5.3	5.8	7.1	3.6	1.35–7.5
Lymphocytes (×10^9^/L)	1.1	0.49	1.0	1.21	3.0	3.0	1.5–4.3
Monocytes (×10^9^/L)	0.26	0.19	0.19	0.15	0.2	0.69	0.25–1.0
Platelets (×10^9^/L)	280	158	111	129	198	254	155–435
Creatinine (µmol/L)	70	76	68	83	80	93	46–96
Urea (mmol/L)	5.1	10.7	9.1	10.9	7.3	6.5	2.5–7.5
Sodium (mmol/L)	136	138	131	134	139	141	135–147
Potassium (mmol/L)	4.7	5.0	4.6	4.2	4.0	3.8	3.5–5.0
Chloride (mmol/L)	98	102	95	98	94	101	98–111
Phosphate (mmol/L)	0.99	0.94	0.95	0.81	—	1.28	0.8–1.4
Bilirubin (µmol/L)	3.6	3.5	9.1	3.0	3.3	2.5	0.0–21
ALT (U/L)	20	42	50	61	56	64	10–45
AST (U/L)	42	27	41	32	40	42	10–45
CK (U/L)	81	—	—	29	72	113	24–195
Aldolase (U/L)	—	6.5	—	—	—	—	0–7.6
LDH (U/L)	358	—	—	467	—	498	135–214
TSH (mU/L)	0.87	—	—	—	—	4.5	0.27–4.2
Cholesterol (mmol/L)	3.29	—	—	—	—	4.9	<5.2
HbA1c (%)	5.8	—	—	—	—	5.7	<6.5
Ferritin (µg/L)	428	1,468	1973	915	722	304	13–150
CRP (mg/L)	10.7	0.3	0.8	0.3	2.1	4.6	0–5.0
ESR (mm/h)	44	5	—	—	106	10	0–20
C3 (g/L)	1.2	—	—	1.1	1.3	1.2	0.9–1.8
C4 (g/L)	0.2	—	—	0.2	0.2	0.2	0.1–0.4
IgG (g/L)	16.9	—	—	12.1	13.9	10.3	7–16
IgA (g/L)	1.74	—	—	1.51	1.65	1.33	0.7–4.0
IgM (g/L)	1.3	—	—	0.91	1.32	0.8	0.4–2.3
IgE (kU/L)	73.6	—	—	—	—	—	5–500
Flow cytometry		October **23**	October **30**				
T Lymphocytes							
CD3^+^ (/µl)	1,169	332	528	1,271	2,666	2,323	782–2,834
CD3^+^CD4^+^ (/µl)	918	264	465	1,047	2,314	1891	322–1750
CD3^+^CD8^+^ (/µl)	241	68	66	225	335	413	338–1,086
CD4^+^/CD8^+^ (/µl)	3.8	3.9	7.0	4.5	6.8	4.6	0.8–2.4
B Lymphocytes							
CD19^+^(/µl)	56	76	201	361	709	527	67–555
NK cells							
CD56^+^CD16^+^ (/µl)	20	5	10	17	27	106	100–645

Immunologic tests revealed anti-nuclear antibodies at 1:160 dilution, with a speckled pattern on HEp-2 cells, anti-Jo-1 antibodies twice, 51 and 50.5 U, respectively (reference value < 20) and a positive direct Coombs’ test. Additional tests for myositis-specific and myositis-associated autoantibodies, *i.e.,* PL-7, PL-12, EJ, OJ, SRP, Mi-2, TIF1γ, MDA5, NXP-2, SS-A, SS-B, PM/Scl-100, U1 RNP, U2 snRNP, U3 RNP, Ku (Mayo Clinic Laboratory, MyoMarker Panel 3, RDL Reference Laboratory, Los Angeles, CA 90034), were negative. The shift in antigenicity from Mi-2 detected in 2011 to Jo-1 was best explained by “epitope spreading” (Cornaby et al., 2015). Other autoantibodies, including anti-dsDNA, anti-Sm, anti-Scl 70, anti-citrullinated peptides, rheumatoid factor, anti-cardiolipin and anti-*β*2-glycoprotein I were undetectable. HLA class II genotyping revealed alleles known to be associated with anti-Jo-1 antibodies in other ethnicities: DRB1*03:01, DRB1*11:04, DQB1*02:01, DQB1*03:01 (Rothwell et al., 2013).

Hematologic abnormalities during the ensuing five-month comprised transient thrombocytopenia (mean 126 ± 16 × 10^9^/L), and monocytopenia (mean 0.17 ± 0.1 × 10^9^/L). In order to derive both percentages and absolute counts of lymphocyte subpopulations flow cytometry was done on heparinized whole blood using BD Multitest™ six-color TBNK reagent and a direct immunofluorescence assay with BD Trucount™ tubes on BD FACSCanto™ II flow cytometer (Becton Dickinson Biosciences, San Jose, CA, United States). At least 5,000 lymphocytes were acquired and analysis was performed using BD FACSDiva™ software version 10.0. Natural killer (NK) cells were defined by analyzing the expression of CD56 and CD16. CD8^+^ T lymphopenia (mean 292 ± 137cells/µl), an increased CD4/CD8 ratio (mean 5.1 ± 1.8), and CD56^+^CD16^+^ NK cytopenia (mean 18.5 ± 11.5 cells/µl) were noted (Orange, 2013; Ebbo et al., 2016). By contrast, counts of CD4^+^ T cells (mean 1,389 ± 650 cells/µl) and CD19^+^ B cells (mean 412 ± 205 cells/µl) fluctuated within normal limits after an initial episode of T and B cell lymphopenia.

### Imaging

**FIGURE 1:**
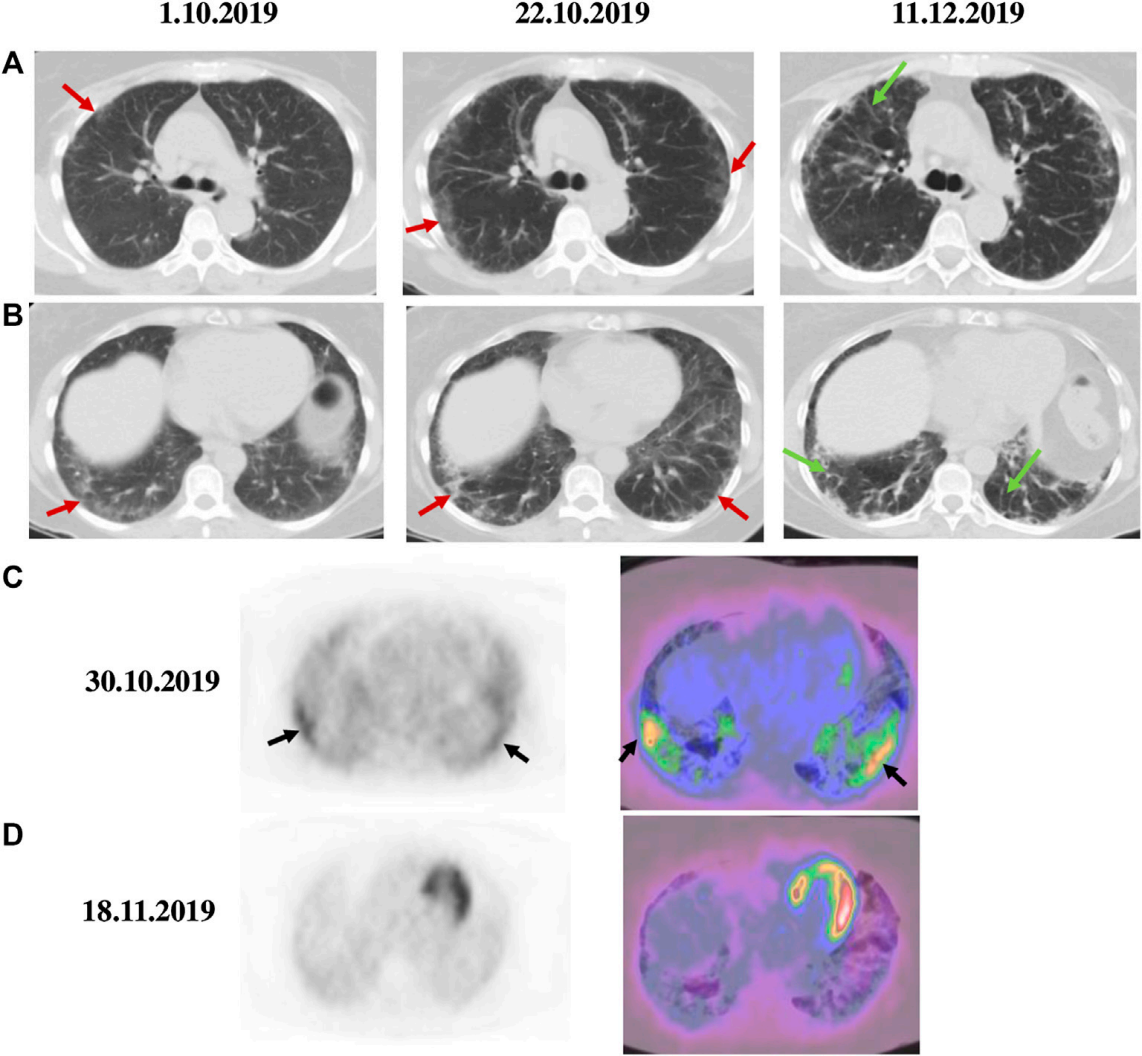
Chest CT and ^18^F-FDG PET/CT scans in anti-Jo-1 antibody-positive RPILD. Serial axial CT scans of upper and middle **(A)** as well as lower lobes of the lung **(B)** showed patchy ground–glass opacities (red arrows) on presentation (October 1, 2019) and worsening on hospital admission (October 22, 2019). Interlobular septal thickening (green arrows) was observed ∼6 weeks after starting therapy with tofacitinib (December 11, 2019). Axial PET **(left)** and fused PET/CT **(right)** images before **(C)** and after three weeks of therapy with tofacitinib **(D)** demonstrated a significant decrease in ^18^F-FDG uptake (SUV max from 4.2 to 1.9) as noted in the lower lung zones (black arrows). Tofacitinib was started on October 30, 2019 and nintedanib added on November 18, 2019.

High resolution (HR) computed tomography (CT) of the chest on presentation showed peripheral ground-glass opacities in both lungs compatible with interstitial pneumonia (Figures 1A,B, October 1, 2019). By echocardiography, no signs of pulmonary hypertension were found.

### Diagnosis

Based upon the clinical presentation, physical examination, pulmonary involvement and newly detected anti-Jo-1 antibodies, an anti-synthetase syndrome (ASS) was diagnosed (Lundberg et al., 2018).

## Therapeutic Interventions and Unanticipated Events

**FIGURE 2:**
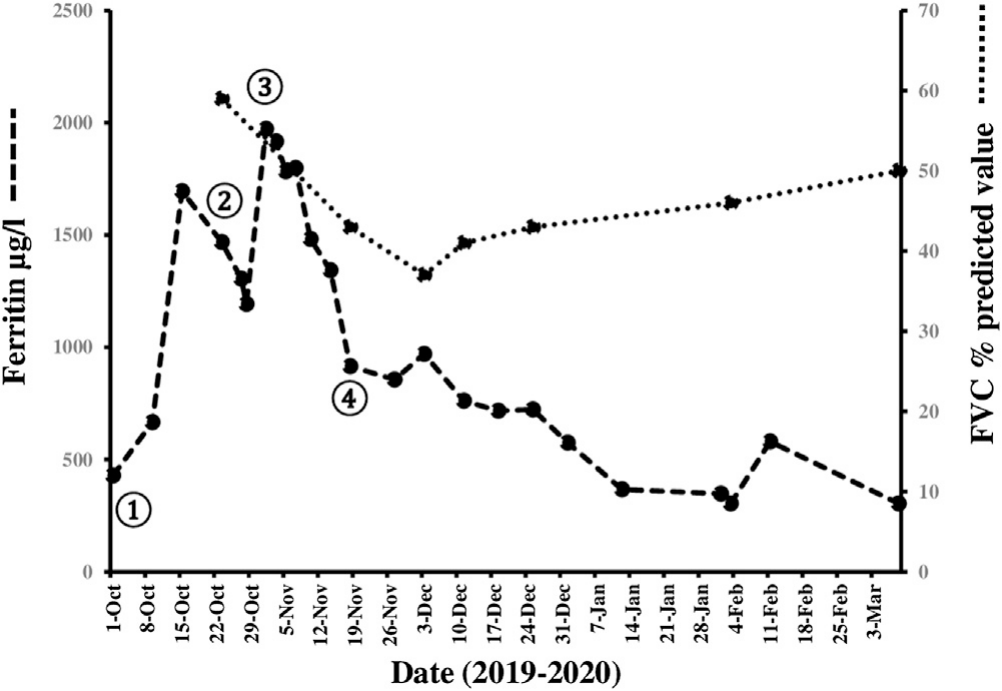
Timeline of pharmacologic interventions, changes in serum ferritin levels and FVC from October 2019 until March 2020. While the patient was treated with high-dose prednisone, azathioprine and tacrolimus ①, the respiratory function suddenly deteriorated and serum ferritin levels (*y*-axis left) rose from 428 to 1,695 μg/L (reference range 13–150 μg/L, dashed line). Pulse therapy with methylprednisolone and IVIG ② were followed by an ephemeral decrease in serum ferritin levels, which rebounded to the maximum measured value of 1973 μg/L. With the administration of tofacitinib first ③ and nintedanib three weeks later ④ we witnessed an immediate clinical improvement and a prompt, continuous decrease in serum ferritin levels to 304 μg/L, which allowed to taper prednisone in short, 10-days intervals. An initial decline of FVC from 59% to 37% was followed by a modest recovery to 50% of predicted value (*y*-axis right; dotted line). Details of doses of the medications used, including the tapering scheme of the corticosteroid, are found in the main text.

The patient was prescribed oral prednisone 80 mg/day, azathioprine 100 mg/day and tacrolimus 1 mg twice a day (Figure 2: ①), with sulfamethoxazole-trimethoprim added for PCP prophylaxis, and followed as an outpatient. While the cutaneous eruptions completely resolved within a three-week period, the respiratory function precipitously and paradoxically deteriorated with dyspnea on minimal exertion, tachypnea of 24 breaths/min and 82% O_2_ saturation in room air requiring continuous O_2_ supplementation of 2–3 L/min with a nasal cannula. The clinical condition required admission to the hospital from October 21, 2019 to November 27, 2019. The pulmonary complication was accompanied by a striking increase in serum ferritin levels from 428 to 1,695 μg/L (Figure 2: *y*-axis left, dashed line). Infections were rigorously ruled out after obtaining broncho-alveolar lavage (BAL) for special stains and cultures through fiber-optic bronchoscopy. The differential cell count of the BAL fluid was as follows: neutrophils 25%, lymphocytes 11%, monocytes 2%, histiocytes 53% and epithelial cells 9%. Chest CT showed expanding bilateral ground-glass opacities (Figures 1A,B, October 22, 2019) and ^18^F-fluorodeoxyglucose (FDG) positron emission tomography (PET)/fused PET/CT scan demonstrated a marked increase in ^18^F-FDG metabolism with standardized uptake value (SUV) max of 4.2 in the lower lung zones and along the pleura with interstitial hyperdensities (Figure 1C). On pulmonary function testing (PFT), the FVC was 59% of predicted value (Figure 2, *y*-axis right).

At this point in time, it was evident that RPILD had independently developed in spite of full control of the cutaneous manifestations. This variant of ILD, which in contrast to our patient typically complicates anti-MDA5 antibody-positive CADM, is notoriously refractory to conventional immunosuppressive regimens and exceptional treatment modalities, such as hemoperfusion with polymyxin B-immobilized fiber column therapy, have been used successfully (Oishi et al., 2013). The patient received initially three doses of methylprednisolone 250 mg daily intravenously (i.v.) and 28 g i. v. immunoglobulins (IVIG) daily on five consecutive days (Figure 2: ②). The serum ferritin level dropped to 1,192 μg/L. However, because her condition remained critical and serum ferritin rebounded to the highest measured level of 1973 μg/L, the decision was taken to initiate treatment with tofacitinib 5 mg twice daily p.o. in combination with methylprednisolone 60 mg/day i.v. on October 30, 2019 (day 1) (Figure 2: ③). Azathioprine and tacrolimus were discontinued.

## Outcomes

**FIGURE 3:**
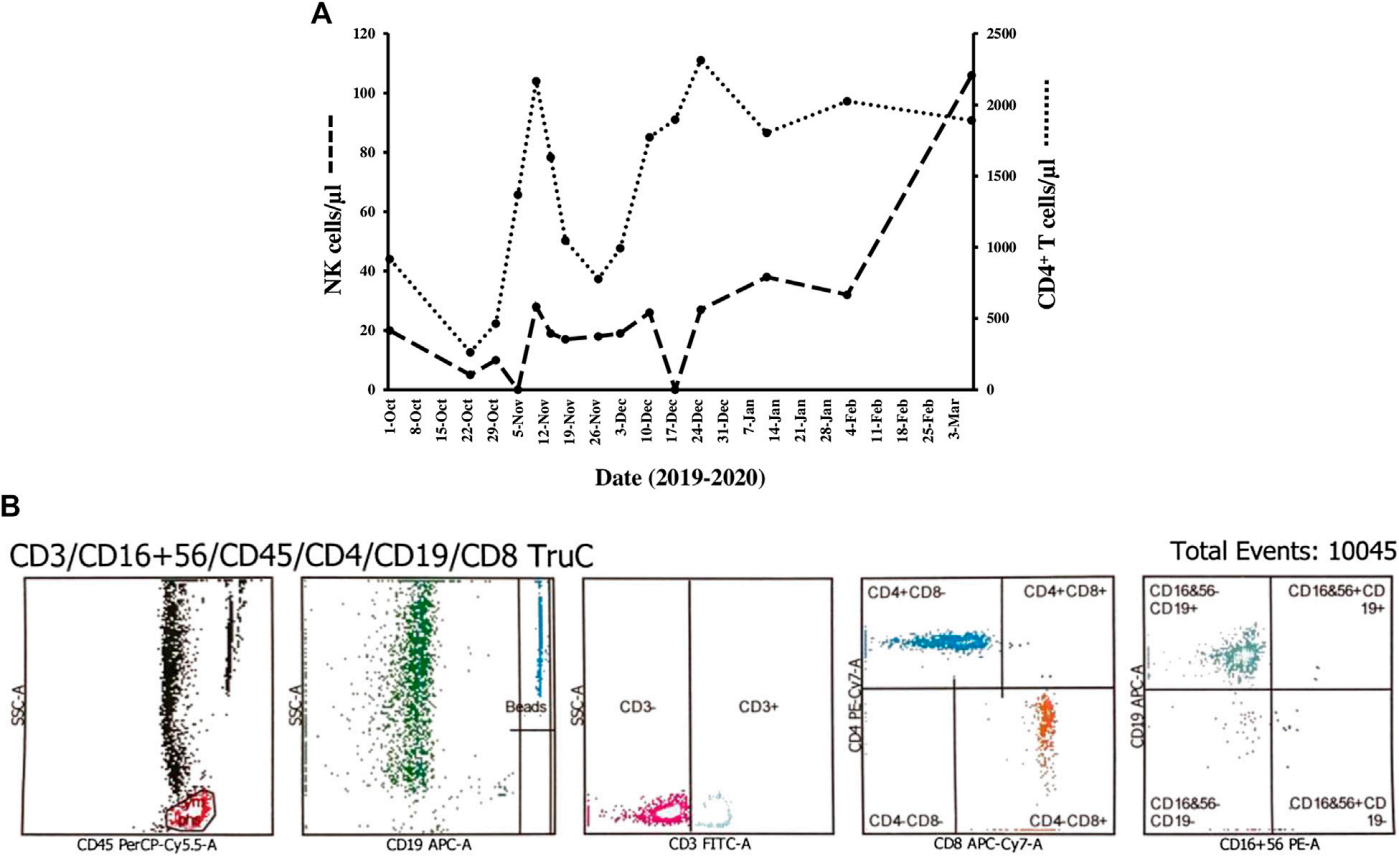
In **(A)**, changes in counts of CD3^−^CD56^+^CD16^+^ NK cells and CD4^+^ T cells are plotted over time. Serial measurements of circulating NK cells/μl (*y*-axis left; dashed line) and CD4^+^ T cells/µl (*y*-axis right; dotted line) by flow cytometry disclosed a persistent, severe numerical NK cell deficiency (mean 18.5 ± 11.5 cells/µl, reference range 100–645 cells/µl), whereas CD4^+^ T cells initially fell to 264 cells/µl on the second day of admission (October 23) and fluctuated considerably within reference range thereafter (mean 1,389 ± 650 cells/µl, reference range 322–1750 cells/µl). NK cell counts returned to normal levels (106 cells/µl) at the end of the observation period. In **(B)**, a representative example of flow cytometry histograms using standard gating strategy is shown. CD45^+^/SSC gate was applied to identify peripheral blood lymphocytes, followed by CD19^+^/SSC, CD3^+^/SSC, CD8^+^ vs. CD4^+^ subsets and finally CD19^+^ vs. NK cells identified as CD3^−^CD19^−^CD56^+^ CD16^+^ lymphocytes, which were nearly absent (histogram #5).

Thereafter, we witnessed an unforeseen, rapid amelioration of respiratory symptoms and parameters, in parallel with an immediate and steady decline in serum ferritin levels to 915 μg/L on November 18, 2019 (day 20) when the pulmonary ^18^F-FDG uptake calculated as SUV max decreased conspicuously from 4.2 to 1.9 (Figure 1D). Since the actual images revealed a reduction in ground-glass attenuation and an increase in interstitial hyperdensities, nintedanib 150 mg twice daily p. o. was added (Figure 2: ④). Prednisolone was substituted for methylprednisolone on day 10 with the following tapering regimen at 10-days intervals: 50–30 (on discharge) -25-20–17.5-15–12.5-10 mg/day. The results of repeated PFT on November 20 (day 22), December 1 (day 33), December 11 (day 43), December 25, 2019 (day 57), February 3 (day 97) and March 9, 2020 (day 132) for FVC in percentage of predicted value were 43%, 37%, 41%, 43% 46% and 50%, respectively (Figure 2: dotted line). A chest CT performed on day 43 after initiating therapy with tofacitinib revealed a reduction in ground-glass opacities and more prominent interlobular septal thickening (Figures 1A,B, December 11, 2019). During this initial observation period, the combination of both kinase inhibitors was safe. Serial measurements of increasing cytomegalovirus (CMV) DNA copies required treatment with oral valganciclovir 900 mg twice daily, which eventually lead to an undetectable viral load. We noted a mild form of macrocytic anemia with hemoglobin concentrations ranging from 95 to 108 g/L and doubling of alanine aminotransferase (ALT) from baseline levels. No gastrointestinal side effects occurred. The patient continued to improve and on her last visit on March 9, 2020 (day 132) vital signs were as follows: temperature 36.7 °C, heart rate 96 beats/min, respiratory rate 20/min, blood pressure 148/81 mmHg and 97% O_2_ saturation in room air. Serum ferritin levels fell further from 717 μg/L (day 50) to 575 μg/L (day 64), 367 μg/L (day 75) and 304 μg/L (day 132). At the end of the reported period, the hematologic abnormalities, including the marked NK cell deficiency, were corrected (Figure 3A: *y*-axis left, dashed line), except for the CD4/CD8 ratio, which remained raised at 4.6. Currently, the patient takes oral prednisone 5 mg/day, tofacitinib 5 mg b.i.d. and nintedanib 150 mg b.i.d.

## Discussion

This case report accurately describes the dramatic sequence of clinical events, laboratory findings and radiographic features in a woman with anti-Jo-1 antibody-positive, anti-MDA5 antibody-negative RPILD, and corroborates the efficacy and safety of tofacitinib followed by the addition of nintedanib later in the management of an aggressive ILD with poor prognosis.

### Distinctive Features in Our Patient With anti-Jo-1 Antibody-Positive RPILD

The individual presented here differs from those reported from Asia not only because of the lack of the anti-MDA5 antibody but also in several other aspects and provides useful information in order to understand the pathogenesis and timeline of changes in various parameters after starting JAK inhibition. First, she represents to our knowledge the first reported case of classic RPILD in anti-Jo-1 antibody-positive DM outside China and Japan treated successfully with tofacitinib. Second, her age was more advanced than the mean age of previously reported cases (74 vs. 47.6 ± 13.8 years) (Chen et al., 2019). Third, RPILD occurred independently despite full control of the inflammatory skin disease while on a combination of corticosteroids, azathioprine and tacrolimus. Fourth, serum ferritin levels are not only an excellent prognosticator, but also provide an ideal biomarker to assess disease activity as well as response to inhibition of JAK signaling. Fifth, a severe, and hitherto unreported, deficiency of CD3^−^CD56^+^CD16^+^ NK cells was observed in the peripheral blood throughout the five-month observation period, which completely reversed on the last visit, suggesting an important role of NK cells in the pathogenesis. Sixth, in parallel to the NK cell deficiency, a decrease in CD8^+^ T cell counts was present, which accounted for a long-lasting increase in the CD4/CD8 ratio. Seventh, ^18^F-FDG PET/fused PET/CT scans allowed not only to localize regions of avid ^18^F-FDG uptake as imaging marker of inflammation but were also capable of detection and quantification of the therapeutic response as early as three weeks after starting treatment with tofacitinib. Eighth, the rate by which we were able to taper (methyl) prednisolone from 60 mg/day indicates that tofacitinib with its immediate onset of action accounts for a potent steroid-sparing effect permitting thereby a faster dose reduction compared to other conditions with critical organ dysfunction. Ninth, we surmise that the early addition of nintedanib at a point in time when inflammation subsided may inhibit the progression into fibrosis. Tenth, the combination of tofacitinib and nintedanib in a 74-year old woman was well tolerated. However, CMV reactivation without evidence of viral illness as observed in other studies required anti-viral treatment (Kurasawa et al., 2018).

### Role of NK Cells, CD4^+^ T Cells and Ferritin in the Pathogenesis of anti-Jo-1 Antibody-Associated RPILD

The paradoxical outbreak of an acute inflammatory interstitial lung disease with no evidence for an infectious etiology coincided with several perturbations of hematologic parameters, the most prominent of which was a severe, yet reversible, quantitative deficiency of CD3^−^CD56^+^ CD16^+^ NK cells in the peripheral blood, first detected when the patient presented. It persisted throughout the flare of the disease and resolved at the end of the reported period. Hypothetically, the striking numerical abnormality was complemented at the functional level by the combination of prednisolone and a calcineurin inhibitor - tacrolimus in our case -, which are both capable of reducing the cytotoxic function of NK cells (Thum et al., 2008; Morteau et al., 2010). The participation of NK cells in the disease process seems plausible as dysfunctional NK cells were identified not only in ASS but also in a variety of other autoimmune and inflammatory disorders with lung involvement, such as Sjoegren’s syndrome, systemic sclerosis, Behcet’s disease, sarcoidosis, hypersensitivity pneumonitis and idiopathic pulmonary fibrosis (Hervier et al., 2019). In patients with end-stage ASS undergoing lung transplantation, it was reported that the fibrotic lung parenchyma of usual interstitial pneumonia (UIP) was massively infiltrated by NK cells with severely impaired synthesis of IFN-γ, whereas counts of NK cells in the peripheral blood were within reference range and similar in controls and diseased (Hervier et al., 2016). Our patient, however, differs from that case series in three main aspects. First of all, she did not have typical features of end-stage UIP such as honeycombing, but RPILD with bilateral, widespread, highly inflammatory infiltrates instead. Second, her ILD was of acute onset with rapid progression while on triple immunosuppression, and third, a severe deficiency of circulating NK cells was persistently present. Therefore, it is conceivable that the pronounced numerical deficiency in NK cells detected in the peripheral blood resulted from redistribution to the lung parenchyma where NK cells with a more pro-inflammatory, *i.e.*, IFN-γ expressing, phenotype could have contributed to the florid inflammation in the lung. Homing of NK cells to target organs takes place in other circumstances as well, *e.g.*, in some infections and autoimmune diseases (Maghazachi, 2010; Zimmer et al., 2019). In support of the assumption of migration of NK cells to the lung parenchyma was the reversibility of this phenomenon after approximately four months of therapy with tofacitinib when counts of NK cells rebounded to >100 cells/µl for the first time.

In parallel to the striking NK cell deficiency, a modest decrease in CD8^+^ T cell counts was noted that accounted for an increase in CD4/CD8 ratios. In addition, we felt that CD4^+^ T cell counts were disproportionally elevated considering the high doses of corticosteroids used. This impression could be related to the recent identification of a distinct CD4^+^CXCR4^+^ T cell subpopulation in Chinese patients with anti-MDA5 antibody-positive RPILD, which correlated with severity and prognosis (Wang et al., 2019). This newly characterized T helper cell subset was considered pathogenic based upon the increased expression of IL-21 and IL-6. IL-21 may act in combination with IL-18 as an additional inducer of the synthesis of ferritin (Dodds et al., 2009; Gono et al., 2012; Slaats et al., 2016). Whether an expanded pro-inflammatory subset of T helper cells and redistributed NK cells could have additively or synergistically elicited the organ threatening pulmonary inflammation is an attractive hypothesis. Moreover, it is worthwhile noting that the heavy chain of ferritin binds specifically to C-X-C motif chemokine receptor (CXCR) 4 and acts as a regulator of the stroma derived factor C-X-C motif ligand (CXCL) 12/CXCR4 signaling pathway suggesting that high ferritin levels, the biochemical hallmark of RPILD, modulate the responses of CD4^+^CXCR4^+^ T cells (Li et al., 2006).

### Tofacitinib’s Unabated *Momentum* in the Acute Management of DM-Associated RPILD and Its Potential to Halt Fibrosis in Combination With Nintedanib

The sequential combination of two kinase inhibitors acting on distinct signaling pathways in lymphoid/myeloid cells on one hand and in mesenchymal cells on the other hand proved to be efficacious and safe in an elderly, non-Asian woman with anti-Jo-1 antibody-associated RPILD confirming that JAK-dependent pathways are critically involved in the pathogenesis of RPILD irrespective of the specificity of the myositis-specific autoantibody. Efficacy encompassed improvement of the respiratory function, radiographic changes, reduction of hyperferritinemia, correction of NK cell deficiency, and reversal of ^18^F-FDG hypermetabolism. Although it is untimely to determine whether structural changes in the lung can be prevented by the combination of tofacitinib and nintedanib, our observations - with the obvious limitations of a solitary case - complement those of others and strongly favor single and possibly dual kinase inhibition as a novel treatment strategy in a condition where conventional immunosuppression has invariably failed. It seems that tofacitinib has “crossed the Rubicon” aiming for the conquest of DM-associated RPILD. Properly designed clinical trials are urgently needed to determine whether tofacitinib or other JAK inhibitors combined or not with nintedanib will become standard of care for the management of acute lung injury related to some subtypes of DM and possibly other immune-mediated lung diseases that are refractory to classic treatment modalities.

## Patient Perspective

As treating physicians, we were extremely concerned when our patient developed severe respiratory distress despite being treated with a classic combination of three immunosuppressants. This was even more disturbing to the patient and her family as she had experienced a complete resolution of the cutaneous manifestations within a short period of time. The patient understood that her health condition was rapidly deteriorating as she became more and more short of breath particularly when she walked short distances. Knowing the prognosis of this particular lung disease and anticipating a worst case scenario at one point in time it was discussed whether she should be considered for lung transplantation. However, recently published data from China and Japan showing the efficacy of tofacitinib gave us hope that this medication would help her condition, and therefore, the patient and family agreed that we should administer the drug as last pharmacological option to intervene. Risks and benefits were extensively discussed. The near instantaneous response to the treatment initiated brought immediate relief to her shortness of breath and ameliorated the irritating cough. Our patient was always cooperative and had full trust in the treating team. Her behavior was exemplary and instrumental to her recovery. She is now extremely grateful and appreciative for the care offered to her knowing that the drug she received was used in a limited number of cases only and that a favorable response was not granted.

## Data Availability Statement

The original contributions presented in the study are included in the article, further inquiries can be directed to the corresponding authors.

## Ethics Statement

The studies involving human participants were reviewed and approved by Research Administration Council (RAC # 2200307), King Faisal Specialist Hospital and Research Centre, Riyadh, Saudi Arabia. The patient provided her written informed consent to participate in this study.

## Author Contributions

WC collected the clinical and immunologic data, reviewed the literature, conceptualized and wrote the manuscript. MK, IW, AA and EAM were responsible for the collection and description of the pulmonary data. MEA performed, interpreted and described the imaging studies. YA took care of the dermatologic aspects. Analysis of flow cytometry was done by NB. WC, MK and IW followed the patient while in hospital and as outpatient. IW helped in the figure design. All authors reviewed and edited the manuscript.

## Conflict of Interest

The authors declare that the research was conducted in the absence of any commercial or financial relationships that could be construed as a potential conflict of interest.

## Abbreviations

ASS, anti-synthetase syndrome; CA, clinically amyopathic; CK, creatine kinase; CMV, cytomegalovirus; CXCL, C-X-C motif chemokine ligand; CXCR, C-X-C motif chemokine receptor; DM, dermatomyositis; FDG, fluorodeoxyglucose; FVC, forced vital capacity; IFN, interferon; ILD, interstitial lung disease; IVIG, intravenous immunoglobulins; JAK, Janus kinase; MCP, metacarpophalangeal; MDA5, melanoma differentiation associated gene 5; NK, natural killer; PCP, Pneumocystis jiroveci pneumonia; PET, positron emission tomography; RP, rapidly progressive; SUV, standardized uptake value; UIP, usual interstitial pneumonia.
